# Genome-wide transcriptional response to silver stress in extremely halophilic archaeon *Haloferax alexandrinus* DSM 27206^ T^

**DOI:** 10.1186/s12866-023-03133-z

**Published:** 2023-12-04

**Authors:** Doriana Mădălina Buda, Edina Szekeres, Lucian Barbu Tudoran, Julia Esclapez, Horia Leonard Banciu

**Affiliations:** 1 Doctoral School of Integrative Biology, Faculty of Biology and Geology, Babeș-Bolyai University, Cluj-Napoca, Romania; 2 Department of Molecular Biology and Biotechnology, Babeș-Bolyai University, Cluj-Napoca, Romania; 3 Institute of Biological Research Cluj, NIRDBS, Cluj-Napoca, Romania; 4 Centre for Systems Biology, Biodiversity and Bioresources, Babeș-Bolyai University, Cluj-Napoca, Romania; 5 National Institute for Research and Development of Isotopic and Molecular Technologies, Cluj-Napoca, Romania; 6 Biochemistry and Molecular Biology and Soil and Agricultural Chemistry Department, Biochemistry and Molecular Biology Area, Faculty of Science, University of Alicante, Alicante, Spain; 7 Emil G. Racoviță Institute, Babeș-Bolyai University, Cluj-Napoca, Romania

**Keywords:** Halophilic archaea, Heavy metal tolerance, RNA-Seq, Silver nanoparticles

## Abstract

**Background:**

The extremely halophilic archaeon *Haloferax (Hfx.) alexandrinus* DSM 27206^ T^ was previously documented for the ability to biosynthesize silver nanoparticles while mechanisms underlying its silver tolerance were overlooked. In the current study, we aimed to assess the transcriptional response of this haloarchaeon to varying concentrations of silver, seeking a comprehensive understanding of the molecular determinants underpinning its heavy metal tolerance.

**Results:**

The growth curves confirmed the capacity of *Hfx. alexandrinus* to surmount silver stress, while the SEM–EDS analysis illustrated the presence of silver nanoparticles in cultures exposed to 0.5 mM silver nitrate. The RNA-Seq based transcriptomic analysis of *Hfx. alexandrinus* cells exposed to 0.1, 0.25, and 0.5 mM silver nitrate revealed the differential expression of multiple sets of genes potentially employed in heavy-metal stress response, genes mostly related to metal transporters, basic metabolism, oxidative stress response and cellular motility. The RT-qPCR analysis of selected transcripts was conducted to verify and validate the generated RNA-Seq data.

**Conclusions:**

Our results indicated that *copA,* encoding the copper ATPase, is essential for the survival of *Hfx. alexandrinus* cells in silver-containing saline media. The silver-exposed cultures underwent several metabolic adjustments that enabled the activation of enzymes involved in the oxidative stress response and impairment of the cellular movement capacity. To our knowledge, this study represents the first comprehensive analysis of gene expression in halophillic archaea facing increased levels of heavy metals.

**Supplementary Information:**

The online version contains supplementary material available at 10.1186/s12866-023-03133-z.

## Background

High salt environments of anthropogenic (e.g. salt crystallizer ponds) or natural origin (e.g. inland endorheic saline lakes) are prone to incidental accumulation of pollutants such as heavy metals due to industrial activities [1]. As these extreme habitats are actively populated by salt-loving bacteria and archaea, the study of interactions between metals and living cells pertaining to halophilic prokaryotes may be beneficial to the current endeavour of developing novel strategies for mitigating metal pollution in saline milieus [2]. Metal-induced stress response in members of bacteria has been intensively studied, revealing various strategies such as extracellular or intracelular metal sequestration, enzymatic detoxification, and/or active efflux of the contaminating metals [3]. To date, however, little information is available on analogous mechanisms in archaea and even lesser in halophilic archaeal representatives [4]. Massive parallel high-throughput sequencing techniques enable the analysis of gene expression dynamics during transcriptomic studies, specifically in response to environmental variations and specific gene factors [5, 6]. Previous studies of prokaryotes exposed to heavy metals revealed a genome-wide response, manifested by differential expression of a wide range of transcripts with functional roles at different cellular levels [7, 8].

Silver ions interact with catalytic thiol groups, form reactive oxygen species (ROS), and induce oxidative stress, in addition to adhering to the cell membrane and compromising its permeability or integrity [9–12]. In response, reduced absorption, ATPase-mediated efflux, and intracellular sequestration are the primary mechanisms employed by living cells for overcoming silver toxicity [13]. The study of the genome-wide transcriptional adjustments during silver stress may link genomic data to biological processes and molecular networks. This approach can potentially unveil crucial gene regulatory targets that play significant roles in promoting heavy metal tolerance [14].

*Haloferax (Hfx.) alexandrinus* is an extremely halophilic archaeon within *Halobacteria* class particularly acknowledged for its ability to synthesize various compounds of significant biotechnological value [15]. Due to its straightforward growth requirements and capacity to thrive in simple media, *Hfx. alexandrinus* demonstrates great adaptability for efficient screening of various metabolic capabilities through high-throughput techniques [16, 17]. Despite its industrial and pharmaceutical potential, the molecular mechanisms underlying its remarkable metabolic flexibility are poorly understood. While *Haloferax volcanii*, a closely related archaeal model, has been extensively explored in physiological and molecular genetics studies [18–20], our focus on *Hfx. alexandrinus* stems from the urge to unravel the unique metabolic adaptations and, in particular, its capacity for silver tolerance, observed in both its type strains and isolated strains. This choice was substantiated by our laboratory observations and corroborated by previous research [21, 22].

The present research employed the type strain *Haloferax alexandrinus* DSM 27206^ T^ as a model organism to explore the cellular components that potentially enable survival in the presence of silver ions. For this purpose, the whole transcriptomes of *Hfx. alexandrinus* DSM 27206 cultures exposed to varying levels of silver were sequenced. Through RNA-Seq data analysis, unique patterns of gene expression variation were revealed among diverse gene sets, encompassing various components of metabolic pathways.

## Methods

### Archaeal strain and cultivation conditions

The investigated strain, *Haloferax (Hfx.) alexandrinus* DSM 27206^ T^, was obtained from the German Colection of Microorganisms and Cell Cultures (DSMZ). The strain was cultivated on the DSMZ medium M372 (Halobacterial medium) containing (g l^−1^): yeast extract, 5; casamino acids, 5; NaCl, 200; MgSO_4_ × 7H_2_O, 20; Na-Glutamate, 1; KCl, 2; Na_3_-citrate, 3; FeCl_2_ × 4H_2_O, 0.036; MnCl_2_, 0.36 × 10^–3^, pH 7 ± 0.2, measured at 25ºC.

The capacity of *Hfx. alexandrinus* to withstand silver was tested by cultivation in liquid M372 media with 0.1, 0.25 and 0.5 mM silver nitrate. The selection of silver nitrate concentrations for testing was guided by previous studies on the silver tolerance of the investigated species [21, 22]. The objective was to detect a discernible response to silver at the lowest concetration, while ensuring an adequate biomass yield by avoiding excessive inhibition of cellular growth at the highest concentration. The stock 1 M silver nitrate (Sigma-Aldrich, St. Louis, MO, USA) solution was filter sterilized and stored at 4ºC, in the dark. To follow the growth, 2 ml aliquots were withdrawn from 30 ml liquid cultures (including control culture with no silver nitrate) every 12 h over 4 days, and growth was monitored by optical density measurements at 623 nm (OD_623_), according to Buda et al. [23]. The results represent the average of three independent experiments, with standard deviations shown as error margins. The specific growth rate was determined according to the methodology outlined by Berney et al*.* [24].

For DAPI (4′,6-diamidino-2-phenylindole) staining, 2 ml aliquots were withdrawn from 30 ml liquid cultures every 12 h over a 4 day period, and cell-count was preformed by dilluting each aliquote with the appropiate volume of ultrapure water, fixing it in 2% glutaraldehide (v/v), and filtering it through 0.22 mm pore size, black-gridded MCE membrane filters (Fioroni, Ingré, France). Subsequently, cells retained on 0.22 mm filters were directly stained using a 5 mg/ml DAPI solution and examined under the microscope Zeiss Axio Scope A1 (Carl Zeiss, Göttingen, Germany).

### Scanning Electron Microscopy coupled to Energy Dispersive Spectrometry analysis (SEM–EDS)

To observe silver nanoparticles, the cells grown for 72 h at highest silver nitrate concentration (0.5 mM) were investigated by Scannning Electron Microscopy with Energy Dispersive X-ray Spectroscopy). Cells were filtered through a 0.45 μm Millipore membrane that was placed on conductive double side carbon tape. After drying, the specimen was sputter coated with gold in an argon atmosphere (AGAR Auto Sputter-Coater, Agar Scientific, Stansted, UK), reaching a final 10-nm thickness. The micrographs were obtained using the scanning electron microscope (Hitachi SU 8230, Tokyo, Japan) and the EDS spectra were generated with the X-ray detector of the same equipment (Oxford Instruments, Abingdon, UK).

### Genomic sequencing

Total genomic DNA (gDNA) was extracted from *Hfx. alexandrinus* cells, cultivated on the recommended DSMZ medium M372, using the Quick-DNA Faecal/Soil Microbe Microprep Kit (Catalog No. D0612, Zymo Research, Orange, CA, USA), starting with 2 ml of a stationary-phase culture (OD_623 nm_ ~ 2). The elution was performed in 30 µl DNA Elution Buffer, the obtained gDNA sample was vaccum-dried using the Savant DNA 120 SpeedVac Concentrator (ThermoFisher Scientific, Waltham, MA, USA). Total gDNA was subjected to paired-end short read sequencing at Novogene Ltd (Hong Kong) on the Illumina HiSeq 2500 platform with a target output of 1 Gbp. De novo genome assembly was executed using the SPAdes assembler with default parameters [25]. Gene prediction and annotation was performed with Prodigal v2.6.3 in genome mode [26], gene annotation was done using KEGG [27], and gene mapping was accomplished by BBMap [28]. The genome statistics were acquired by employing custom scripts along with the Prokka software [29], while the completeness and contamination of the assembled genome were assessed using CheckM v1.1.0 [30]. Furthermore, the identification of differentially expressed transcripts in the RNA-Seq dataset were carried out by mapping pairs of short RNA-Seq reads to predicted genes from the genome.

### RNA extraction

Transcriptomic analysis by RNA-Seq was performed in triplicate on *Hfx. alexandrinus* grown at 0, 0.1, 0.25 and 0.5 mM silver nitrate. The cultures were prepared by inoculating an early stationary phase pre-culture (OD_623 nm_ ≈ 1.6) in 75 ml M372 media containing the corresponding silver nitrate concentrations, followed by incubation at 110 rpm and 37ºC*.* The time point for RNA isolation was chosen following the recommendation of using mid-exponential phase cells for RNA extractions [31, 32], but also to guarantee accumulation of sufficient biomass within cultures with delayed growth. Therefore, the biomass was collected once cultures reached mid-exponential phase (OD_623 nm_ ~ 0.7—1), as follows: the control cultures after 16 h, the 0.1 and 0.25 mM silver nitrate-grown cultures after 22 h, and the 0.5 mM silver nitrate-grown cultures after 28 h. The cultures were centrifuged at 5 000 × g, for 5 min, the pellet was resuspended in 1.5 ml of the obtained supernatant, followed by a second centrifugation at 6 000 × g, for 3 min [33]. The collected pellet was further employed for total RNA extraction, using the Direct-zol RNA MiniPrep Kit (Catalog No. R2052, Zymo Research, Orange, CA, USA), with the following modification: prior to RNA extraction according to the standard kit protocol, the cells were processed with 23-gauge needles and syringes to guarantee complete cell lysis [33]. The obtained RNA was spectrophotometricaly assessed by Nanodrop spectrophotometer ND-1000 (Thermo Scientific, Waltham, MA, USA) and electrophoresis on a denaturing agarose gel (1% w/v). Control no-challenge culture replicates were processed in the same manner without the addition of silver nitrate, and the samples were further stored at -80ºC until future analyses. A quantity of 5 µg RNA from each sample was vaccum-dried (Savant DNA 120 SpeedVac Concentrator, Thermo Scientific, Waltham, MA, USA) in RNAStable (Biomatrica, Inc., San Diego, CA, USA). RNA-Seq analysis was performed at Novogene Ltd (Hong Kong) following the procedure described below.

### RNA-Seq analysis

The study involved quality-controlled RNA samples depleted in rRNA using the Ribo-Zero Magnetic Kit (Epicenter, Madison, WI, USA), followed by random fragmentation and reverse transcription employing the mRNA-template. The second cDNA strand was synthesized using a custom second-strand synthesis buffer (Illumina Inc., San Diego, CA, USA), dNTPs, RNase H, and DNA polymerase I. Double-stranded cDNA libraries (strand specific, 250–300 bp insert) were fed into the Illumina NovaSeq 6000 sequencer, with raw Illumina PE150 sequencing data filtered to remove adaptors, reads containing more than 10% uncertain nucleotides, or reads with more than 50% low quality nucleotides. For the alignment, the Bowtie2 v2.3.4.3 algorithm [34] was employed, using the *Hfx. alexandrinus* DSM 27206^ T^ genome as reference, while the mismatch parameter was set to two, and other parameters were set to default. Gene expression levels, measured by transcript abundance, were examined using the FeatureCounts v1.5.0-p3 software [35] and were expressed as FPKM (fragments per kilobase of transcript per million fragments mapped) values. The differential gene expression (DEG) analysis was performed using the DESeq2 software [36]. Additionally, the enrichment analysis of the differentially expressed genes was performed using the clusterProfiler [37] program and the gene sets provided by the KEGG database.

### Validation of RNA-Seq results by RT-qPCR

The RNA-Seq results were validated by the reverse transcription quantitative PCR (RT-qPCR) analysis of the identical RNA specimens, employing a total of 10 genes, for which the gene-specific primers utilized are presented in Additional file 1 [7, 38]. The 16S rRNA gene copies were used as internal reference, employing previously verified *Haloferax*-specific primers [39]. Following the DNase treatment with the TURBO DNA-free kit (ThermoFisher Scientific, Waltham, MA, USA), single stranded cDNA was prepared from the extracted RNA using random hexameric primers, as well as the RevertAid First Strand cDNA Synthesis kit (ThermoFisher Scientific, Waltham, MA, USA). cDNA levels were quantified using a Bio-Rad CFX Real Time System (BioRad, Hercules, CA, USA). The program consisted of an initial denaturation at 95 °C for 180 s, followed by 40 cycles of 15 s at 95 °C and an annealing/elongation step performed at 60 °C for 60 s. The reaction mixtures contained the following components: 5 μl SYBR Green Mix, 0.3 μM of the forward and reverse primers, 0.5 μl cDNA, and RNase/DNase-free water to a final volume of 10 μl. For reaction specificity assessments a post-PCR melting curve analysis was performed, in which the temperature ramped between 65 °C and 95 °C in 0.5 °C increments with subsequent plate readings. The values recorded at logarithmic phase represent the average of three biological replicates, each comprising eight technical replicates. The relative expression of each transcript was calculated using the standard 2^−ΔΔCt^ formula [40], with the 16S rRNA gene as the reference. To assess the correlation between the fold-chage data from RNA-Seq and RT-qPCR results, Pearson’s correlation coefficient was calculated using GraphPad Prism 9 (GraphPad Software, San Diego, CA). A statistically significant difference was determined by considering a *P*-value < 0.05.

## Results and discussion

### Effect of different silver nitrate concentrations on *Haloferax alexandrinus* cell growth

**Fig. 1:**
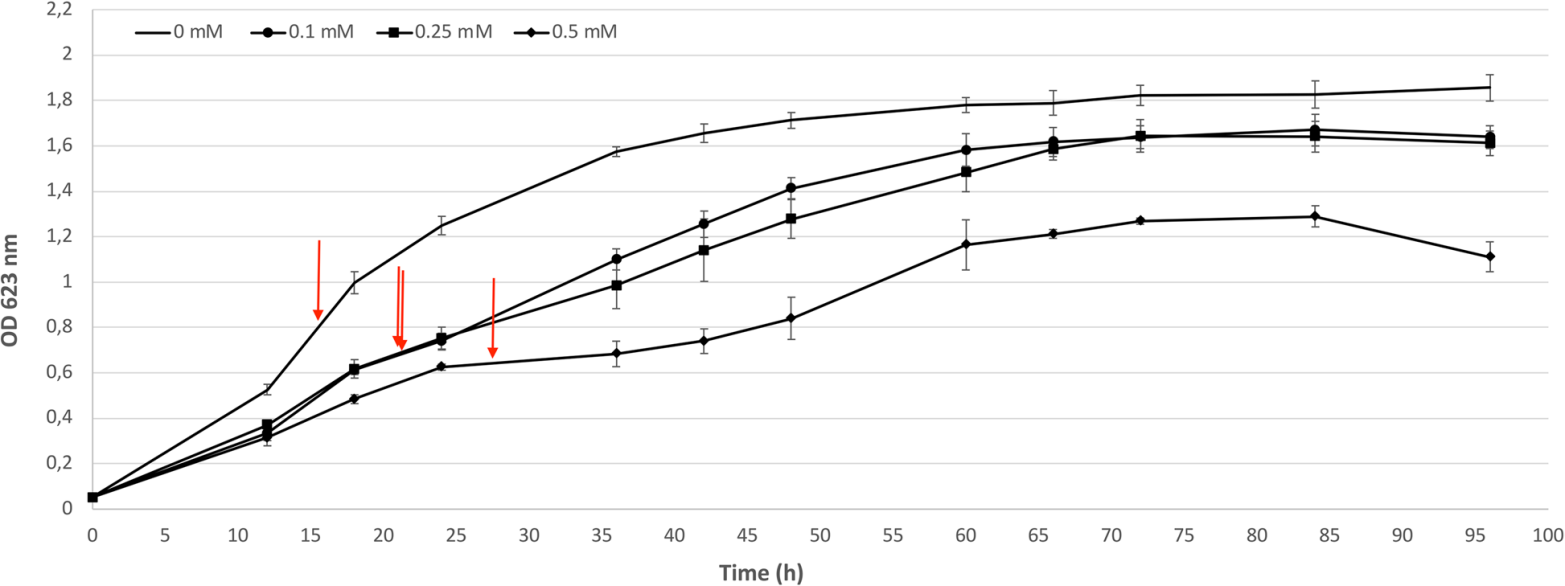
Growth curves of cells cultured in M372 medium or in M372 supplemented with 0.1, 0.25, or 0.5 mM silver nitrate. The arrows indicate the point in time at which the biomass for the transcriptomic analysis was collected. The values are means for triplicate cultures and the error bars indicate standard deviations

**Fig. 2:**
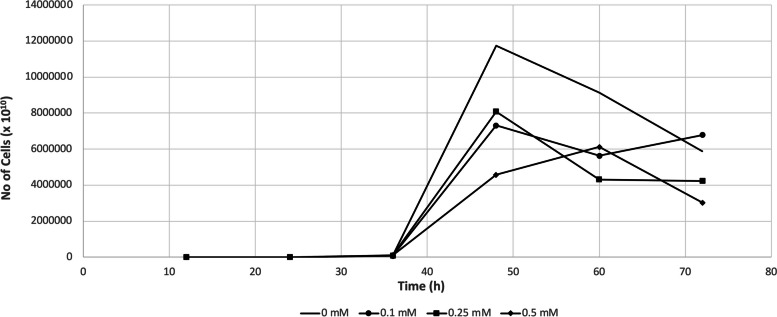
DAPI-staining cell-count of *Hfx. alexandrinus* cultured in M372 medium or in M372 supplemented with 0.1, 0.25, or 0.5 mM silver nitrate

The growth of *Hfx. alexandrinus* was monitored in increasing silver nitrate concentrations ranging from 0 to 0.5 mM by OD_623 nm_ and DAPI staining cell-count, during 96 h, and 72 h (Figs. 1 and 2). When compared to the control cultures (0 mM silver nitrate), the cultures grown at 0.1 to 0.5 mM silver nitrate showed a lagging period and a slightly lower specific growth rate: 0.083 h^−1^ for the control cultures, followed by 0.076, 0.074 h^−1^, and 0.064 h^−1^ for cells cultivated with 0.1, 0.25, and 0.5 mM silver nitrate, respectively. These results suggest that, while the concentration of silver nitrate increased, the rate of cell multiplication declined, as cells were constraint to redistribute their resources to ensure survival [41].

**Fig. 3:**
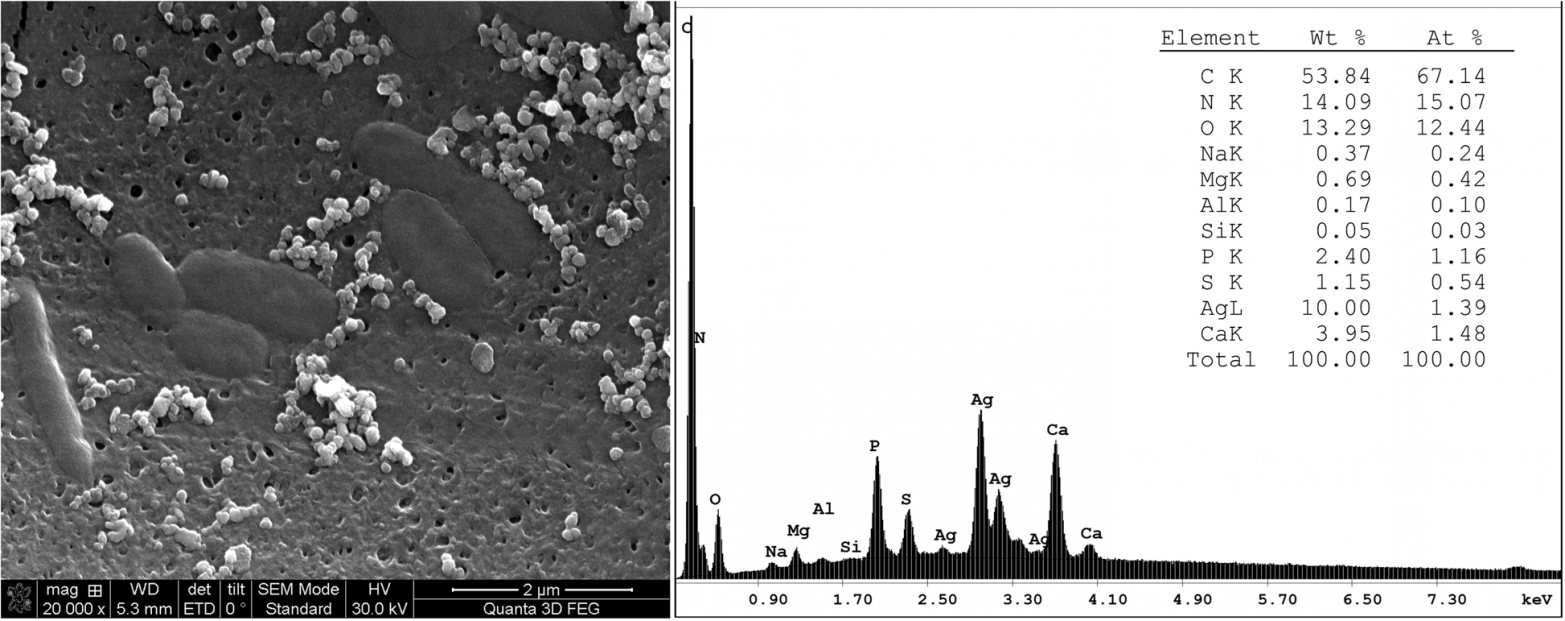
SEM–EDS analysis of *Hfx. alexandrinus* cells incubated with 0.5 mM silver nitrate for 72 h. Left panel: micrograph of silver-exposed cells. Right panel: the elemental composition of haloarchaeal cells surrounded by silver nanoparticles

The SEM–EDS analysis of *Hfx. alexandrinus* cells grown at highest silver concentrations (0.5 mM silver nitrate) revealed the presence of extracellular silver-containing nanoparticles (Fig. 3). This observation aligns with prior studies that have delved into the physiological mechanisms underpinning silver resistance and the potential involvement of nanoparticle biosynthesis by *Hfx. alexandrinus* [21, 22]. The results suggest a plausible connection between nanoparticle biosynthesis and the cellular response to metal stress, emphasizing a potential mechanism for metal tolerance in this organism.

### Genome characteristics of *Haloferax alexandrinus* DSM 27206^ T^

The sequenced genome of *Hfx alexandrinus* DSM 27206^ T^ displayed a GC content of 66.21% and a total length of 3,670,276 base pairs across 31 contigs. It contains 3,668 coding sequences (CDS) with an average length of 268.11 amino acids, resulting in a coding density of 86.53%. Noncoding regions accounted for 13.47% of the genome, with a mean intergenic spacer length of 135.75 base pairs. Quality assessment confirmed a high completeness of 99.57%, and low contamination of 0.92% for the *Hfx. alexandrinus* DSM 27206^ T^ genome. The estimated coverage indicated a depth of sequencing at 324x, underscoring the robustness of the data. This data was supported by similar investigations of related strains (*Haloferax* sp. ATB1, *Hfx. volcanii* DS2, *Hfx. denitrificans*, *Hfx. mucosum*, and *Hfx. sulfurifontis*) yielding genome sizes of 3–4 Mbp distributed among 21–120 contigs, with GC contents of 61–66%. These sequenced genomes exhibited coverage levels of 25-224x, and contained over 3,000 coding sequences [18, 42, 43].

### Overview of transcriptome features in the silver-exposed cells

**Fig. 4:**
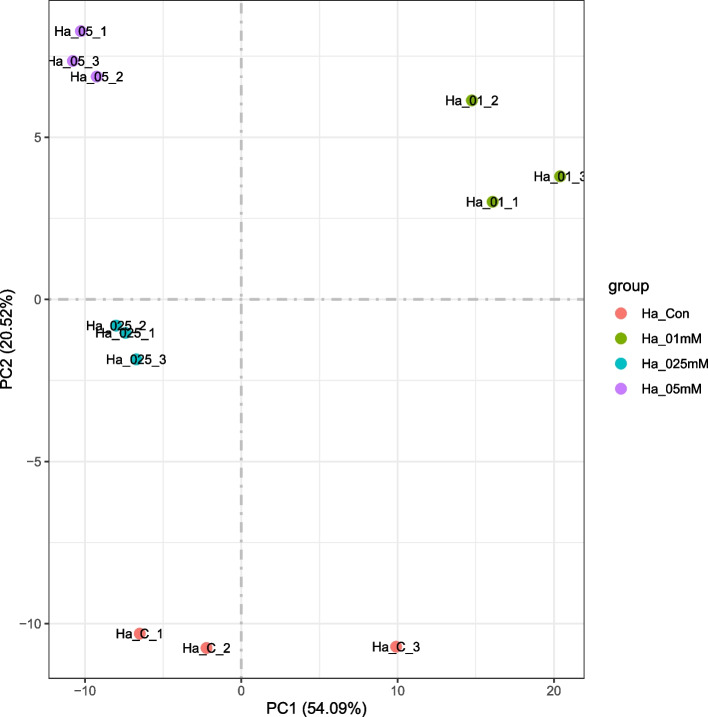
PCA (principal component analysis) of the correlation between the RNA-Seq samples. Biological triplicates of the samples are included, as follows: Ha_C_1, Ha_C_2, and Ha_C_3- control cultures; Ha_01_1, Ha_01_2, and Ha_01_3- cells cultured with 0.1 mM silver nitrate; Ha_025_1, Ha_025_2, and Ha_025_3- cells cultured with 0.25 mM silver nitrate; Ha_05_1, Ha_05_2, and Ha_05_3- cells cultured with 0.5 mM silver nitrate

A number of 13.6 to 22 million RNA-Seq reads were generated for each sample, with an average total mapping rate of 82.52% to the *Hfx. alexandrinus* DSM 27026^ T^ genome (See Additional file 1). Principal Component Analysis (PCoA) revealed greater dissimilarities between transcriptional profiles altered by experimental conditions than among biological replicates (Fig. 4).

**Fig. 5:**
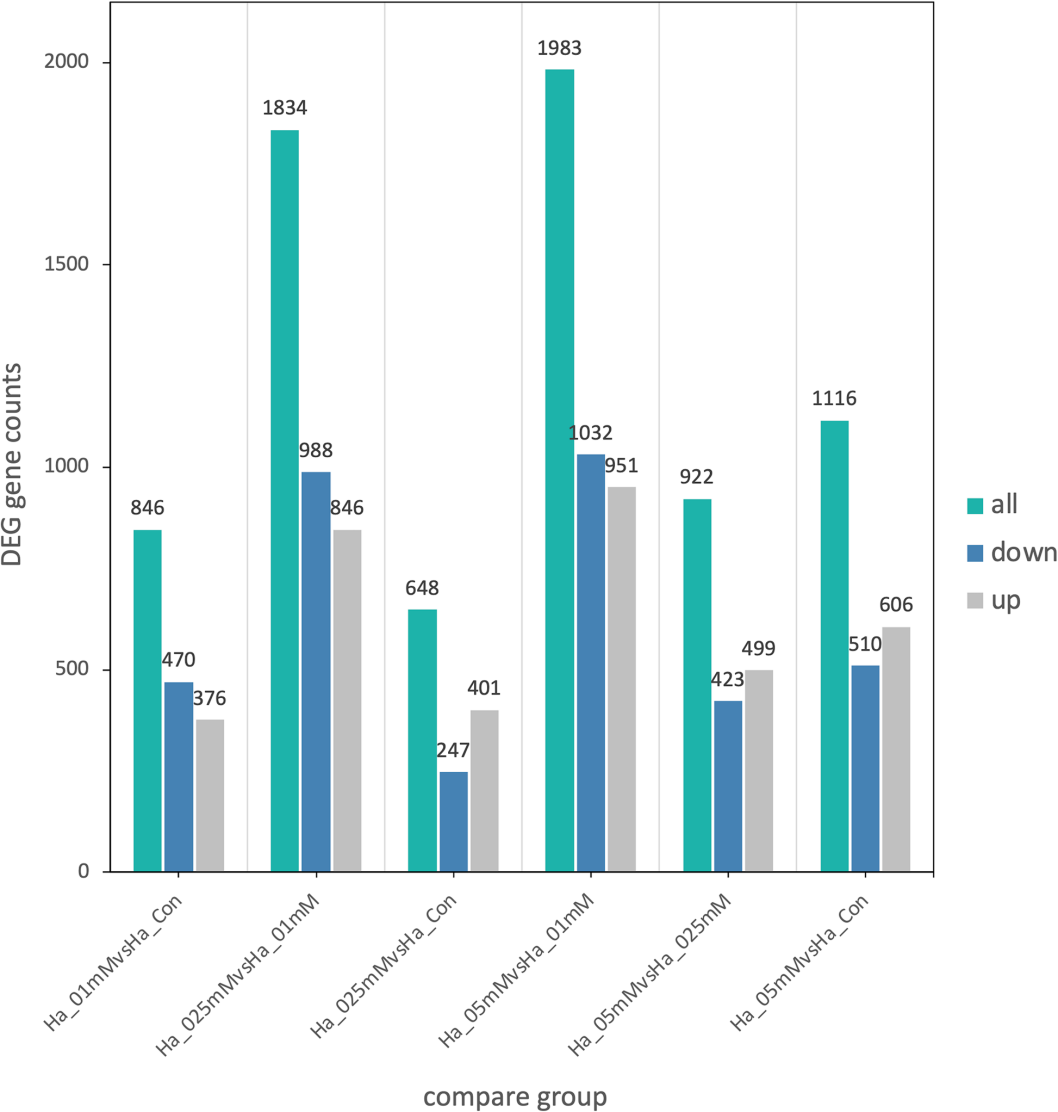
The statistical summary of the differential gene expression analysis results. The threshold for differentially expressed genes (DEGs) is: padj < 0.05; |log_2_FoldChange|> 0.0. Ha_Con: control cultures; Ha_01mM: cells cultured with 0.1 mM silver nitrate; Ha_025mM: cells cultured with 0.25 mM silver nitrate; Ha_05mM: cells cultured with 0.5 mM silver nitrate

The highest number of genes (1116) affected by growth conditions was detected in culture grown at 0.5 mM silver nitrate whereas cells grown at 0.1 and 0.25 mM silver nitrate showed 846 and 648 up- or downregulated genes, respectively (Fig. 5).

**Fig. 6:**
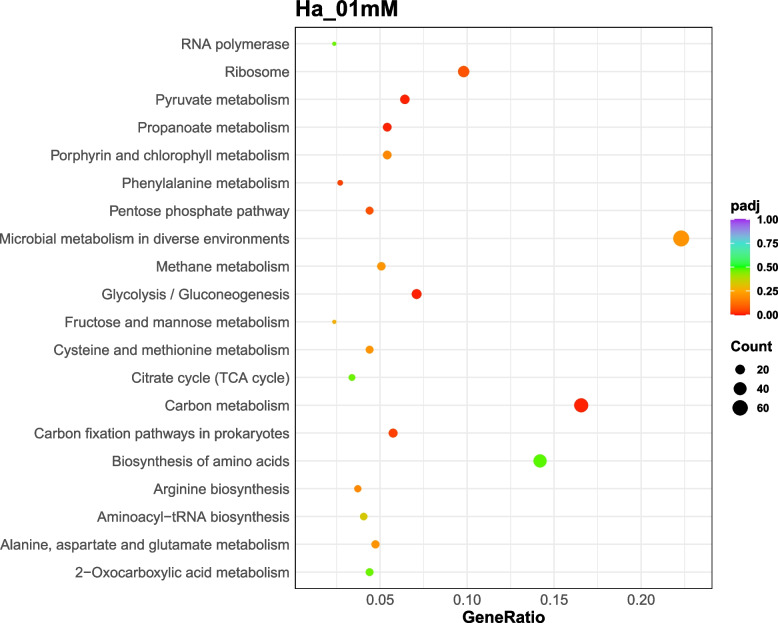
KEGG enrichment scatter plot of DEGs in Ha_01mM (cells cultured with 0.1 mM silver nitrate) vs Ha_Con (control cultures). The y-axis represents the name of the pathway, while the x-axis illustrates the Rich factor, calculated as the ratio of the number of DEGs to the total number of genes assigned to a certain pathway. Each dot in the plot has a size that corresponds to the number of different genes, and a color that represents the matching q-value. Pathways showing corrected *p*-values < 0.05 exhibit a notable enrichment in differentially expressed genes (DEGs). The enrichment becomes more significant as the q-value approaches zero

**Fig. 7:**
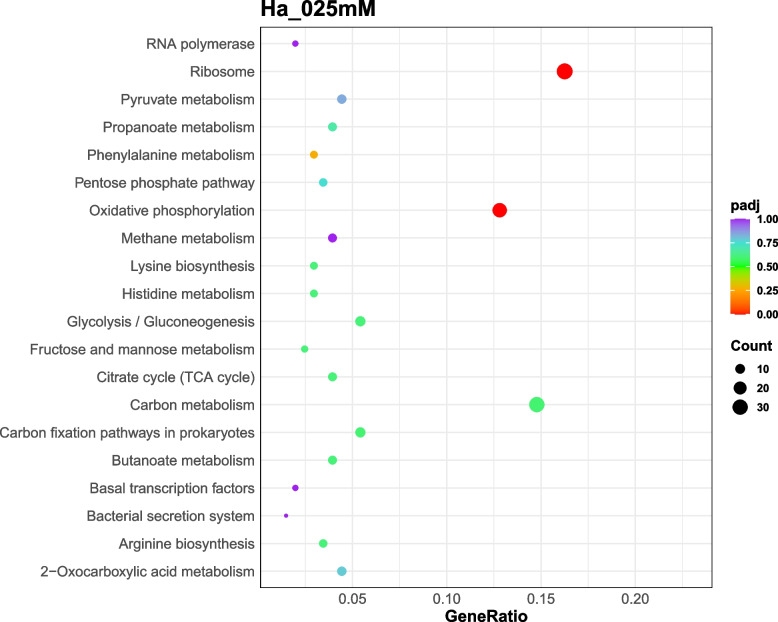
KEGG enrichment scatter plot of DEGs in Ha_025mM (cells cultured with 0.25 mM silver nitrate) vs Ha_Con (control cultures). The y-axis represents the name of the pathway, while the x-axis illustrates the Rich factor, calculated as the ratio of the number of DEGs to the total number of genes assigned to a certain pathway. Each dot in the plot has a size that corresponds to the number of different genes, and a color that represents the matching q-value. Pathways showing corrected *p*-values < 0.05 exhibit a notable enrichment in differentially expressed genes (DEGs). The enrichment becomes more significant as the q-value approaches zero

**Fig. 8:**
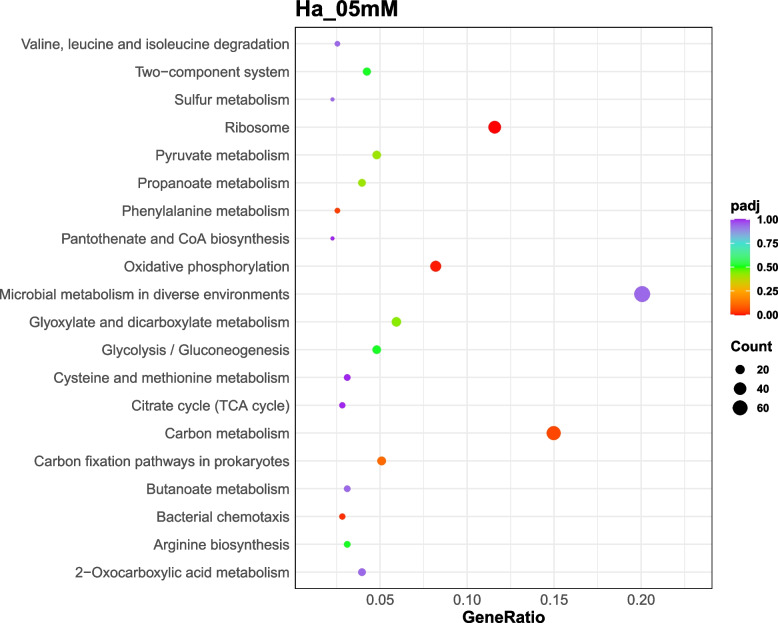
KEGG enrichment scatter plot of DEGs in Ha_05mM (cells cultured with 0.5 mM silver nitrate) vs Ha_Con (control cultures). The y-axis represents the name of the pathway, while the x-axis illustrates the Rich factor, calculated as the ratio of the number of DEGs to the total number of genes assigned to a certain pathway. Each dot in the plot has a size that corresponds to the number of different genes, and a color that represents the matching q-value. Pathways showing corrected *p*-values < 0.05 exhibit a notable enrichment in differentially expressed genes (DEGs). The enrichment becomes more significant as the q-value approaches zero

The enrichment analysis of differential expressed genes provided insights into the biological functions and pathways altered by silver stress in *Hfx. alexandrinus*. Thus, exposure to 0.1 mM silver induced modifications in 130 genes associated with basic cellular metabolism, whilst exposure to 0.25 mM silver led to the alterations of 33 genes associated with genetic information processing, and 26 genes related to energy metabolism. Likewise, the presence of 0.5 mM silver nitrate induced changes in 33 genes linked to basic cellular metabolism, 41 genes associated with genetic information processing, and 10 genes related to cellular processes (Figs. 6, 7, 8). The functions of hypothetical proteins remain poorly understood up to date, and their roles in silver tolerance are not discussed here.

### Metal transporters and metal-related genes

Our study revealed that the copper transporter gene *copA* (Hfx-2019-SC1-cds4) was strongly induced under all three experimental conditions (Table 1). Conversely, exposure to higher silver concentrations resulted in the upregulation of the transcriptional regulator *lrp* (Hfx-2019-SC1-cds5) known to be involved in the resistance to metallic ions, as well as the copper chaperone *copZ* (Hfx-2019-SC1-cds6). CopZ, with its versatile protein folds, functions as metal-binding units for the transport of copper, silver, cadmium, and mercury [44].

**Table 1:** Transcription rates of metal transporters and metal-related genes^a^

*Hfx. alexandrinus* DSM 27206 ORF^b^	Gene name	Log_2_FC			Annotation
		0.1 mM	0.25 mM	0.5 mM	
Hfx-2019-SC1-cds4	*copA*	3.25	4.48	5.64	Cu^+^-exporting ATPase
Hfx-2019-SC1-cds5	*lrp*	-	2.2	3.73	Transcriptional regulator
Hfx-2019-SC1-cds6	*copZ*	-	-	3.1	Copper chaperone
Hfx-2019-SC1-cds149	*petE*	5.49	-	-	Plastocyanin/azurin family
Hfx-2019-SC1-cds430		3.14	-	-	HTH_ARSR domain containing protein
Hfx-2019-SC3-cds305		-	-	2.09	HTH_ARSR domain containing protein
Hfx-2019-SC4-cds254		2.08	-	2.67	ABC.PE.A1 peptide/nickel transport system ATP-binding protein
Hfx-2019-SC4-cds255		-	-	2.84	ABC.PE.A peptide/nickel transport system ATP-binding protein
Hfx-2019-SC4-cds257		-	-	2.84	ABC.PE.P peptide/nickel transport system permease protein
Hfx-2019-SC4-cds258	*ddpA*	-	-	2.19	ABC.PE.S peptide/nickel transport system substrate-binding protein
Hfx-2019-SC4-cds326	*petE*	-	-	3.06	Plastocyanin/azurin family
Hfx-2019-SC5-cds117	*mntH*	-	-	2.43	Manganese transport protein
Hfx-2019-SC4-cds164	*mobA*	-	-	-5.72	Molybdopterin-guanine dinucleotide biosynthesis protein A
Hfx-2019-SC4-cds165	*wtpA*	-	-	-5.94	Molybdate/tungstate transport system substrate-binding protein
Hfx-2019-SC4-cds166	*wtpB*	-	-	-5.15	Molybdate/tungstate transport system permease protein
Hfx-2019-SC4-cds220		-	-2.68	-	LucA/LucC family protein member
Hfx-2019-SC4-cds224	*fepB*	-2.17	-	-	ABC.FEV.S iron complex transport system substrate-binding protein
Hfx-2019-SC4-cds239	*cibT*	-2.16	-	-	Cobalt-precorrin-6B (C15)-methyltransferase
Hfx-2019-SC4-cds240	*cbtB*	-2.9	-	-	Cobalt transporter subunit, putative
Hfx-2019-SC4-cds241	*cbtA*	-2.83	-	-	Cobalt transporter subunit, putative
Hfx-2019-SC4-cds243	*asnC*	-2.09	-	-	Lrp/AsnC family transcriptional regulator
Hfx-2019-SC4-cds337		-2	-	-	ABC.PE.P1 peptide/nickel transport system permease protein

^a^RNA-Seq was used to determine the expression levels of the genes in *Hfx. alexandrinus* DSM 27206, as detailed in the section on Materials and Methods. The data are the average of three replicates. The threshold of differential expression genes was: |log_2_(FoldChange)|> 1 and qvalue < 0.00. Dashes indicate that there were minimal changes to the transcription, not considered statistically significant. ^b^The gene name, number, and annotation were generated through the gDNA sequencing of the tested strain, as described in the Materials and Methods section

CopA was identified as an exporting ATPase that is responsible for silver detoxification in *Sulfolobus solfataricus* [45]. Due to its high silver affinity, another P_1B_-type ATPase, CopB, was additionally suggested for the silver resistance of *S. solfataricus*; however, this transporter is not encoded by genome of *Hfx. alexandrinus* DSM 27206^ T^. Evidence from *Enterococcus hirae* suggests that the interaction between CopZ and CopA enhances the elimination of internal copper by transferring it to related proteins: as the level of cytoplasmic copper rises, CopZ serves as a chaperone that selectively transports Cu to the CopY repressor [46–48].

The Lrp/AsnC family of transcriptional factors has been described as one of the most common archaeal global and local transcription regulators that mediate survival in harsh conditions, including metal-induced stress [41, 49, 50]. In *Hfx. alexandrinus,* based on its chromosomal location and relationship with the transcription of *copA* and *copZ*, Lrp may regulate their transcription in response to silver toxicity.

Similar to the Cu-related gene cluster of the studied strain, the *copA* operon was previously identified in the silver-resistant strains including *S. solfataricus*, consisting of *copA*, a copper binding protein (*copT*), and the regulator (*copR*) [48, 51, 52]. The significant upregulation of the CopA ATPase accompanied by two additional copper-related proteins strongly suggests that the mechanism underlying silver tolerance in *Hfx. alexandrinus* is mostly dependent on CopA-mediated efflux [12]. These results strengthen the hypothesis brought forward by Liu et al*.* [53], suggesting that metal transporters act as first line of defense against acute exposure to metals and are crucial components of the rapid response system that control the movement of metal ions within cells.

Two HTH-ASR domain-containing transcriptional repressors from the SmtB/ArsR family, Hfx-2019-SC1-cds430 and Hfx-2019-SC3-cds305, were upregulated in response to 0.1 mM and 0.5 mM silver nitrate (Table 1). As effect of silver ions, Hfx-2019-SC1-cds430 may regulate the expression of nearby genes, including a probable plastocyanin/azurin family member.

At 0.1 mM and 0.5 mM silver nitrate, two *petE* (Plastocyanin/azurin family member) genes, Hfx-2019-SC1-cds149 and Hfx-2019-SC4-cds326 were strongly induced by 5.49 and 3.06 log_2_FC (Table 1). A protein that resembles plastocyanins and is believed to be involved in the production of the proton motive force was previously identified in *Hfx. volcanii* [54, 55]. However, as noted by Llorca et al*.* [56], due to the lack of plastocyanin-like genes in non-photosynthetic organisms, the genes may have been mistakenly annotated in the case of *Haloferax mediterranei*. Indeed, the blastp query of the two proteins from *Hfx. alexandrinus* DSM 27206^ T^ against the NCBI non-redundant protein database indicated that they were related to the family of copper-binding proteins called plastocyanin/azurins. The discovery that CinA homologs are frequently linked to CopA, raises the possibility of an electron transfer function [57]. Moreover, several studies have demonstrated that copper homeostasis is significantly impacted by cellular components that are abundant in thiol groups, particularly in *Halothiobacillus* [58, 59].

Peptide/nickel transport system ATP-binding proteins (Hfx-2019-SC4-cds254 and Hfx-2019-SC4-cds255), substrate-binding protein (Hfx-2019-SC4-cds258), and permease protein (Hfx-2019-SC4-cds257) were upregulated under silver ions, along with *mntH* (manganese transport protein, Hfx-2019-SC5-cds117)- Table 1. Previous studies demonstrated similar overexpression patterns of related genes in response of microbial communities to Pb [60] and *Pseudomonas putida* to zinc exposure [7].

At 0.25 mM, the transcription of a LucA/LucC family protein member (according to blastp results), Hfx-2019-SC4-cds220, was downregulated by -2.68 log_2_FC (Table 1). The LucA/LucC family proteins were assigned in *Hfx. mediterranei* and cyanobacteria as iron-dependent siderophore biosynthesis proteins that support non-specific Cu import [56, 61]. Therefore, downregulation of these proteins that could promote silver absorption might act as a barrier preventing silver ions from entering cells.

### Oxidative stress and energy metabolism

In all silver nitrate-treated cultures, the NhaC Na^+^:H^+^ antiporter (Hfx-2019-SC1-cds370) exhibited the highest level of gene induction among non-metallic transporters (Table 2). The protein plays a crucial role in maintaining pH homeostasis in prokaryotes, by increasing proton import and reducing cytoplasmic alkalinization [62, 63]. In halophilic archaea, NhaC aids Na^+^ extrusion during osmotic upshock [64], while it also supports the extrusion of other cations (e.g., Li^+^) in *Bacillus sp.* [65, 66]. Moreover, NhaC was up-regulated in *Pseudomonas putida*, in response to As and thiocyanate-induced alkaline stress [67]. These antiporters also contain USP (Universal Stress Protein)- specific domains, that contribute to cell resilience during challenging circumstances, including oxidative stress [68–70]. In silver-treated *Hfx. alexandrinus*, NhaC may function as a protective mechanism against heavy metal toxicity by regulating intracellular pH homeostasis and maintaining osmotic balance.

**Table 2:** Transcription rates of genes involved in the oxidative stress response and energy metabolism^a^

*Hfx. alexandrinus* DSM 27206 ORF^b^	Gene name	Log_2_FC			Annotation
		0.1 mM	0.25 mM	0.5 mM	
Hfx-2019-SC1-cds370	*nhaC*	4.16	2.1	2.31	Na^+^:H^+^antiporter
Hfx-2019-SC1-cds423	*norB*	2.35	-	-	Nitric oxide reductase subunit B
Hfx-2019-SC3-cds277	*cydB*	-	-	2.61	Cytochrome d ubiquinol oxidase subunit II
Hfx-2019-SC2-cds328	*nuoCD*	2.09	-	2.08	NADH-quinone oxidoreductase subunit C/D
Hfx-2019-SC5-cds137		2.25	-	2.07	Flavin-dependent oxidoreductase
Hfx-2019-SC5-cds143	*putA*	3.11	-	2.77	Aldehyde dehydrogenase (NAD^+^)
Hfx-2019-SC2-cds705	*porB*	-2.09	-	-	Pyruvate ferredoxin oxidoreductase beta subunit
Hfx-2019-SC2-cds772				-2.34	COG2210 Peroxiredoxin family protein member
Hfx-2019-SC4-cds7	*narC*	-	-	-4.28	Cytochrome b-561
Hfx-2019-SC4-cds158		-	-	-2.25	4Fe-4S ferredoxin iron-sulfur binding domain-containing protein, N-terminal
Hfx-2019-SC4-cds159		-	-	- 2.15	Prokaryotic molybdopterin-containing oxidoreductase family, binding subunit
Hfx-2019-SC4-cds160	*hybA*	-	-	-2.89	Fe-S-cluster-containing hydrogenase components 1
Hfx-2019-SC4-cds161		-	-	-3.90	Prokaryotic molybdopterin-containing oxidoreductase family, membrane subunit
Hfx-2019-SC4-cds162	*torD*	-	-	-4.79	Uncharacterized component of anaerobic dehydrogenases
Hfx-2019-SC4-cds163		-	-	-5.16	Oxidoreductase, molybdopterin-binding domain containing protein
Hfx-2019-SC4-cds242		- 2.95	-	-	Thioredoxin (TRX)-like [2Fe-2S] Ferredoxin (Fd) familymember

^a^RNA-Seq was used to determine the expression levels of the genes in *Hfx. alexandrinus* DSM 27206, as detailed in the section on Materials and Methods. The data are the average of three replicates. The threshold of differential expression genes was: |log_2_(FoldChange)|> 1 and qvalue < 0.00. Dashes indicate that there were minimal changes to the transcription, not considered statistically significant. ^b^The gene name, number, and annotation were generated through the gDNA sequencing of the tested strain, as described in the Materials and Methods section

Cells exposed to 0.1 and 0.5 mM silver nitrate increased the expression of several energy metabolism genes, including aldehyde dehydrogenase (Hfx-2019-SC5-cds143), and Hfx-2019-SC3-cds277, subunit II of cytochrome *d* ubiquinol oxidase (Table 2). Similar investigations demonstrated that cytochrome *bd* respiratory oxygen reductases were activated in O_2_-limited settings in addition to other potentially stressful growth conditions, such as Fe-deficit [71, 72].

However, silver nitrate-treated cultures displayed decreased levels of several oxidative stress-related transcripts, including components of pyruvate ferredoxin and molybdopterin-containing oxidoreductases, Fe-S-cluster-containing hydrogenase, peroxiredoxins, and electron transport components (Table 2). As thioredoxin fold proteins (thioredoxins, preoxiredoxins, oxidoreductases, etc.) are considered prominent cellular protectors against oxidative stress [73, 74], these results seem rather intriguing and demand further investigations.

### Basic metabolism

#### Carbohydrate metabolism

At 0.1 mM silver nitrate, several carbohydrate metabolism enzymes were upregulated (Table 3), including glyceraldehyde-3-phosphate dehydrogenase (Hfx-2019-SC3-cds294), and pyruvate water dikinase (Hfx-2019-SC2-cds179). Recent studies have characterized two functionally distinct GADPHs in *Hfx. volcanii*: GAPDHI [EC:1.2.1.12], involved in glucose catabolism, and GAPDHII [EC:1.2.1.59], associated with gluconeogenesis [75]. In *Hfx. alexandrinus*, silver nitrate led to the overexpression of GADPHII equivalent, sustaining the biosynthesis of cellular components required to mitigate damage caused by silver ions, while suppressing glycolytic Class I GADPH (Hfx-2019-SC3-cds298), and inducing *ppsA* (phosphoenolpyruvate synthetase)- Table 3. Phosphoenolpyruvate synthetase has also demonstrated to play a role in gluconeogenesis, particularly in the conversion of pyruvate to phosphoenolpyruvate in *Hfx. mediterranei* [76].

**Table 3:** Transcription rates of genes involved in basic metabolism^a^

*Hfx. alexandrinus* DSM 27206 ORF^b^	Gene name	Log_2_FC			Annotation
		0.1 mM	0.25 mM	0.5 mM	
Carbohydrate metabolim					
Hfx-2019-SC1-cds280		-	-	2.23	ABC.SS.P simple sugar transport system permease protein
Hfx-2019-SC1-cds281		-	-	2.22	ABC.SS.P simple sugar transport system permease protein
Hfx-2019-SC1-cds378	*gatC*	2.51	-	-	Galactitol-specific IIC component
Hfx-2019-SC2-cds179	*ppsA*	2.13	-	-	Pyruvate water dikinase
Hfx-2019-SC2-cds823		-	-	3.49	COG0701 Predicted permeases
Hfx-2019-SC2-cds834	*fbp*	3.74	-	-	Fructose-1 6-bisphosphatase I
Hfx-2019-SC3-cds294	*gap2*	2.53	-	-	Glyceraldehyde-3-phosphate dehydrogenase (NAD(P))
Hfx-2019-SC5-cds125	*galE*	3.83	-	3.63	UDP-glucose 4-epimerase
Hfx-2019-SC5-cds135	*ich-Y*	-	-	2.2	Itaconyl-CoA hydratase
Hfx-2019-SC1-cds496	*ugpB*	-	-	-2.01	sn-glycerol 3-phosphate transport system substrate-binding protein
Hfx-2019-SC1-cds980	*iolG*	-2.32	-	-	Myo-inositol 2-dehydrogenase / D-chiro-inositol 1-dehydrogenase
Hfx-2019-SC2-cds552		-	-2.27	-	AAA-type ATPase, core component
Hfx-2019-SC2-cds877	*gnaD*	-2.9	-	-	Gluconate/galactonate dehydratase
Hfx-2019-SC3-cds298	*gapA*	-2.47	-	-	Glyceraldehyde 3-phosphate dehydrogenase
Hfx-2019-SC3-cds336	*gtsA*	-3.46	-	-	Glucose/mannose transport system substrate-binding protein
Hfx-2019-SC3-cds337	*gtsB*	-3.83	-	-	Glucose/mannose transport system permease protein
Hfx-2019-SC3-cds338	*gtsC*	-3.06	-	-	Glucose/mannose transport system permease protein
Hfx-2019-SC3-cds339	*msmX*	-3.78	-	-	Multiple sugar transport system ATP-binding protein
Hfx-2019-SC3-cds350	*araE*	-	-	-2.44	Arabinose:H^+^ symporter
Hfx-2019-SC4-cds36	*iolE*	-	-	-6.43	Sugar phosphate isomerases/epimerases
Hfx-2019-SC4-cds78	*msmX*	-	-2.34	-	Multiple sugar transport system ATP-binding protein
Hfx-2019-SC4-cds146	*thuA*	-	-2.92	-4.28	Trehalose utilization protein
Hfx-2019-SC4-cds211	*gtsC*	-	-	-5.63	Glucose/mannose transport system permease protein
Lipid metabolim					
Hfx-2019-SC2-cds258	*acs*	2.2	-	2.5	AMP-forming acetyl-CoA synthetase
Hfx-2019-SC2-cds631	*acs*	2.39	-	-	AMP-forming acetyl-CoA synthetase
Hfx-2019-SC2-cds779	*caiC*	2.01	-	-	Fatty-acyl-CoA synthase
Hfx-2019-SC5-cds129		2.6	-	-	Enoyl-CoA hydratase
Hfx-2019-SC5-cds130	*paaH*	2.82	-	4.02	3-hydroxybutyryl-CoA dehydrogenase
Hfx-2019-SC5-cds131	*pksG*	3.19	-	3.59	hydroxymethylglutaryl-CoA synthase
Hfx-2019-SC5-cds132	*paaJ*	2.28	-	3.15	Acetyl-CoA C-acetyltransferase
Hfx-2019-SC5-cds133	*paaK*	3.3	-	3.49	Phenylacetate-CoA ligase
Hfx-2019-SC5-cds134	*paaI*	2.86	-	3.83	Acyl-CoA thioesterase
Hfx-2019-SC5-cds136	*epi*	2.14	-	2.16	Methylmalonyl-CoA/ethylmalonyl-CoA epimerase
Hfx-2019-SC5-cds142	*paaI*	2.75	-	2.95	Acyl-CoA thioesterase
Hfx-2019-SC5-cds145	*paaD*	2.9	-	-	Predicted metal-sulfur cluster biosynthetic enzyme
Hfx-2019-SC5-cds146	*paaC*	3.17	-	2.45	Ring-1,2-phenylacetyl-CoA epoxidase subunitPaaC
Hfx-2019-SC5-cds147	*paaB*	3.17	-	2.44	Ring-1 2-phenylacetyl-CoA epoxidase subunitPaaB
Hfx-2019-SC5-cds148	*paaA*	2.69	-	-	Ring-1 2-phenylacetyl-CoA epoxidase subunit PaaA
Hfx-2019-SC5-cds76	*prpD*	-	-4.03	-	2-methylcitrate dehydratase
Hfx-2019-SC5-cds77	*prpD*	-	-3.36	-	2-methylcitrate dehydratase
Amino acid metabolism					
Hfx-2019-SC1-cds368	*puuE*	2.81	-	2.17	4-aminobutyrate aminotransferase
Hfx-2019-SC2-cds36	*pdhC*	2.56	-	-	Pyruvate dehydrogenase E2 component
Hfx-2019-SC4-cds252	*hyuA*	-	-	2.19	N-methylhydantoinases
Hfx-2019-SC5-cds98	*hutH*	2.17	-	-	Histidine ammonia-lyase
Hfx-2019-SC5-cds118	*hyuA*	-	-	2.30	N-methylhydantoinase
Hfx-2019-SC5-cds119	*hyuB*	2.18	-	2.23	N-methylhydantoinases
Hfx-2019-SC5-cds120	*pepQ*	3.56	-	2.11	Xaa-Pro dipeptidase
Hfx-2019-SC5-cds126	*ilvB*	3.59	-	4.02	Acetolactate synthase I/II/III large subunit
Hfx-2019-SC5-cds151		2.1	-	-	Amidohydrolase
Hfx-2019-SC1-cds54	*RP-S6e*	-2.72	-	-	RPS6 small subunit ribosomal protein S6e
Hfx-2019-SC3-cds359	*livK*	-	-	-2.59	Branched-chain amino acid transport system substrate-binding protein
Hfx-2019-SC4-cds106	*ilvA*	-	-2.53	-2.05	Threonine dehydratase
Hfx-2019-SC4-cds221		-	-2.08	-	Aspartate aminotranferase family member
Hfx-2019-SC4-cds222	*dat*	-	-2.99	-2.48	Diaminobutyrate-2-oxoglutarate transaminase
Hfx-2019-SC5-cds223	*pydC*	-	-	-2.05	Beta-ureidopropionase/ N-carbamoyl-L-amino-acid hydrolase
Hfx-2019-SC6-cds119	*lysK*	-2.16	-	-2.15	LysW-gamma-L-lysine/LysW-L-ornithine carboxypeptidase
Hfx-2019-SC6-cds120	*lysJ*	-2.01	-	-	LysW-gamma-L-lysine/LysW-L-ornithine aminotransferase
Nucleotide metabolism					
Hfx-2019-SC7-cds82	*draG*	-	-2.37	-	ADP-ribosylglycohydrolase

^a^RNA-Seq was used to determine the expression levels of the genes in *Hfx. alexandrinus* DSM 27206, as detailed in the section on Materials and Methods. The data are the average of three replicates. The threshold of differential expression genes was: |log_2_(FoldChange)|> 1 and qvalue < 0.00. Dashes indicate that there were minimal changes to the transcription, not considered statistically significant. ^b^The gene name, number, and annotation were generated through the gDNA sequencing of the tested strain, as described in the Materials and Methods section

On the other hand, silver nitrate significantly suppressed the expression of multiple transport proteins, as well as the trehalose utilization protein (Hfx-2019-SC4-cds146), and *myo*-inositol 2-dehydrogenase/D-chiro-inositol 1-dehydrogenase (Hfx-2019-SC1-cds980)- Table 3. In archaea, components like trehalose and di-*myo*-inositol-1-phosphate are essential for modulating osmoadaptation and cellular protection during various stress conditions [77–80]. The accumulation of these molecules occurs when the enzymatic pathways responsible for their degradation are inhibited. This preservation of trehalose and di-*myo*-inositol-1-phosphate in response to silver stress suggests the activation of a nonspecific survival mechanism triggered by exposure to metals.

#### Lipid metabolism

A significant number of components of the phenylacetic acid (PAA) degradation pathway were induced by the presence of silver (*paaABCDHIJK*, Table 3). This pathway plays a crucial role in the bacterial breakdown of aromatic compounds, with PAA serving as a key intermediate metabolite [81]. While halophilic archaea were previously thought to only degrade aromatic compounds using dioxygenases [82], a PAA pathway has been described in *Thermoprofundales* [83]. Recent studies of *Acinetobacter* have revealed a potential link between oxidative stress management and the PAA pathway [81]. These findings imply that the upregulation of the *paa* cluster may contribute to silver tolerance in *Hfx. alexandrinus*. Furthermore, other oxidative stress-related enzymes, located adjacent to the *paa* genes on the *Hfx. alexandrinus* genome, were also upregulated (e.g., Hfx-2019-SC5-cds143, described above). At 0.1 mM silver nitrate, enoyl-CoA hydratase (Hfx-2019-SC5-cds129), a fatty acid-based monomer-supplier, was also upregulated. Previous studies have shown that PaaG, a component of to the PAA pathway, belongs to the enoyl-CoA hydratase/isomerase family [81]. Therefore, considering the co-occurrence of Hfx-2019-SC5-cds129 with the PAA pathway-associated cluster, it suggests its involvement in the enzymatic processes and metabolic transformations related to PAA degradation.

Exposure to 0.1 and 0.5 mM silver nitrate led to the upregulation of AMP-forming acetyl-CoA synthetases (*acs*, Hfx-2019-SC2-cds258 and Hfx-2019-SC2-cds631; Table 3). These enzymes are responsible for the conversion of acetate to acetyl-CoA, a crucial intermediate in various anabolic and catabolic pathways [84]. The observed increase in expression levels may be ascribed to the cellular endeavor to balance energy versus survival in the presence of silver, but also to the previously registered interference of heavy metals with the enzymatic activity of *acs* in *Methanococcus* [85, 86].

Hydroxymethylglutaryl-CoA synthase (HMG-CoA synthase) has been identified as crucial for membrane biogenesis in *Hfx. mediterranei*, where it was found to be upregulated in response to oxidative stress-induced damage [85, 87]. In the present study, we hypothesize that HMG-CoA synthase (Hfx-2019-SC5-cds131; Table 3) may play a similar role in cells of silver-stressed *Hfx. alexandrinus.* However, further investigations are required to determine its specific contribution to membrane biogenesis under these conditions.

#### Amino acids metabolism

At 0.1 and 0.5 mM silver nitrate concentrations, several genes encoding enzymes involved in amino acids turnover were upregulated, including Xaa-Pro aminopeptidase (Hfx-2019-SC5-cds120), 4-aminobutyrate aminotransferase (*puuE*, Hfx-2019-SC1-cds368), and acetolactate synthase, large subunit (Hfx-2019-SC5-cds126) (Table 3). To our knowledge, the role of PuuE, a member of the aspartate aminotransferase protein family in haloarchaea, is not well-understood. However, early studies suggest that pyridoxal phosphate-dependent aspartate aminotransferases catalyze amino group transfer of in *Hfx. mediterranei* [88]. Aminopeptidases and ammonia-lyases may indirectly contribute to the detoxification of heavy metals by reducing the oxidative damage produced by silver, in addition to supporting the preservation of the intracellular reservoir of amino acids acquired from peptides [89, 90].

Among the downregulated genes associated with amino acids metabolism were those encoding the small subunit ribosomal protein RPS6 (Hfx-2019-SC1-cds54), a carboxypeptidase (Hfx-2019-SC6-cds119) and several aminotransferases (Hfx-2019-SC6-cds120, Hfx-2019-SC4-cds221, Hfx-2019-SC4-cds222; Table 3). These proteins could undergo downregulation as part of a physiological response aimed at reducing the overall protein synthesis machinery or controlling the generation/degradation of specific amino acids. This cellular response helps conserve resources and mitigates the accumulation of potentially harmful metal-protein complexes.

#### Nucleotide metabolism

The ADP-ribosylglycohydrolase family members play a crucial role in the ADP-ribosylation pathway, which links and detaches ADP-ribose tags to proteins, nucleic acids, and small molecules. These systems are widespread across all life domains due to frequent gene transfer and are central to various prokaryotic conflict systems, stress responses, DNA damage responses, and antibiotic resistance [91–93]. Nonetheless, given the observed downregulation of the gene encoding ADP-ribosylglycohydrolase (Hfx-2019-SC7-cds82) in silver-exposed *Hfx. alexandrinus* (Table 3), the enzyme may contribute to the compensation of the stress response with low energy metabolism (e.g., through transcription control) in the presence of silver nitrate.

### Motility

Genes encoding for motility-related proteins were predominantly downregulated upon exposure to 0.5 mM silver nitrate (Table 4), suggesting a reduction in cell movement capability. Specifically, genes encoding flagellins *flaB* (Hfx-2019-SC2-cds605) and *flaD/flaE,* as well as chemotaxis proteins such as *mcp* (methyl-accepting chemotaxis protein, Hfx-2019-SC3-cds360), *cheF* (Hfx-2019-SC2-cds616), *cheD* (Hfx-2019-SC2-cds600), and *cheY* (Hfx-2019-SC2-cds602) were suppressed in the presence of silver. Impaired motility in silver-contaminated media could serve as a protective mechanism, inhibiting further metal uptake from the environment and reducing exposure to potentially hazardous levels of heavy metals [94]. Previous studies have shown that a common bacterial stress response to heavy-metal poisoning includes decreased cell motility and chemotaxis in *Caulobacter*, *Shewanella*, and *Bacillus cereus* exposed to silver nitrate [8, 94–96]. In *Haloarcula marismortui*, investigations revealed that the multiple-encoded pilins and archaellins function as ecoparalogs, meaning they are expressed in response to specific environmental conditions, providing adaptive advantages at various salt concentrations [97, 98].

**Table 4:** Transcription rates of genes involved cellular motility^a^

*Hfx. alexandrinus *DSM 27206 ORF^b^	Gene name	Log_2_FC			Annotation
		0.1 mM	0.25 mM	0.5 mM	
Hfx-2019-SC2-cds599	*flaD/flaE*	-	-	-2.02	Flagellar protein family
Hfx-2019-SC2-cds600	*cheD*	-	-	-2.03	Chemotaxis protein
Hfx-2019-SC2-cds602	*cheY*	-	-	-2.01	Two-component system chemotaxis family response regulator
Hfx-2019-SC2-cds605	*flaB*	-	-	-3.39	Archaeal flagellin
Hfx-2019-SC2-cds606	*flaB*	-	-	-3.33	Archaeal flagellin
Hfx-2019-SC2-cds616	*cheF*	-	-	-2.12	Chemotaxis protein
Hfx-2019-SC3-cds360	*mcp*	-	-	-2.32	Methyl-accepting chemotaxis protein

^a^RNA-Seq was used to determine the expression levels of the genes in *Hfx. alexandrinus* DSM 27206, as detailed in the section on Materials and Methods. The data are the average of three replicates. The threshold of differential expression genes was: |log_2_(FoldChange)|> 1 and qvalue < 0.00. Dashes indicate that there were minimal changes to the transcription, not considered statistically significant. ^b^The gene name, number, and annotation were generated through the gDNA sequencing of the tested strain, as described in the Materials and Methods section

### DNA-binding proteins

MC1 (Hfx-2019-SC6-cds1), an archaeal-specific DNA-binding protein involved in genome compaction [99, 100], exhibited upregulation in response to the presence of 0.5 mM silver nitrate (Table 5). Conversely, the MC1 knockout mutant of *Hfx. volcanii* displayed minimal developmental defects and no significant difficulties under various stress conditions [101]. Additionally, several helix-turn-helix domain- containing proteins (Hfx-2019-SC3-cds305, Hfx-2019-SC1-cds430, and Hfx-2019-SC1-cds429) were overexpressed in response to silver exposure (Table 5), suggesting their roles as regulators in the cellular adaptive response to silver-induced stress [102].

**Table 5:** Transcription rates of DNA- binding proteins^a^

*Hfx. alexandrinus *DSM 27206 ORF^b^	Gene name	Log_2_FC			Annotation
		0.1 mM	0.25 mM	0.5 mM	
Hfx-2019-SC1-cds429		2.41	-	-	Helix-turn-helix domain-containing proteins
Hfx-2019-SC1-cds430		3.14	-	-	Helix-turn-helix domain-containing proteins
Hfx-2019-SC3-cds305		-	-	2.09	Helix-turn-helix domain-containing proteins
Hfx-2019-SC6-cds1	*MC1*	-	-	2.02	Non-histone chromosomal protein
Hfx-2019-SC1-cds537	*xerD*	-	-2.49	-2.11	Integrase/recombinase
Hfx-2019-SC1-cds560	*sbc*	-	-3.3	-	Exonuclease
Hfx-2019-SC1-cds580		-	-	-2.01	Orc1-type DNA replication protein
Hfx-2019-SC2-cds231		-	-	-2.27	HNH endonuclease
Hfx-2019-SC7-cds13		-	-4.04	-4.62	DNA-binding protein
Hfx-2019-SC7-cds14		-	-3.56	-3.95	HNH endonuclease
Hfx-2019-SC7-cds48		-	-2.63	-3.21	DNA-binding protein

^a^RNA-Seq was used to determine the expression levels of the genes in *Hfx. alexandrinus* DSM 27206, as detailed in the section on Materials and Methods. The data are the average of three replicates. The threshold of differential expression genes was: |log_2_(FoldChange)|> 1 and qvalue < 0.00. Dashes indicate that there were minimal changes to the transcription, not considered statistically significant. ^b^The gene name, number, and annotation were generated through the gDNA sequencing of the tested strain, as described in the Materials and Methods section.

Silver nitrate suppressed a series of DNA-processing proteins, including exo-/endo- nucleases and an integrase-recombinase, as well as the Orc1-type DNA replication protein (Table 5). Heavy metals induce oxidative stress, leading to DNA damage, and disruption of vital processes such as DNA replication and repair [8]. Consequently, cells downregulate specific DNA-related proteins to delay these processes, preserve genomic integrity and conserve energy. This shift in focus from growth to survival and stress adaptation might help cells prioritize DNA protection over repair processes, similar to the strategy observed in *Rhodobacter* cells exposed to UVB radiation [103].

### Structural cellular components

Under cultivating conditions with 0.25 mM silver, the expression of a predicted membrane protein containing the SHCOT domain (Hfx-2019-SC1-cds84) was induced. The SHOCT sequence has been previously identified as a localization domain in prokaryotes, responsible for anchoring surface proteins of the cell [104]. Moreover, exposure to 0.5 mM silver nitrate induced the upregulation of the basic membrane protein A (Hfx-2019-SC1-cds278), as well as the S-layer domain COG136-containing protein (Hfx-2019-SC5-cds185; Table 6). Previous studies have suggested that the COG1361 domain is involved in the synthesis of the cell membrane and S-layer components in thermoacidophilic archaea [105, 106]. In addition to reinforcing cellular structures, the overexpression of these enzymes can facilitate the production of components responsible for coating and stabilizing the observed extracellular silver nanoparticles [107].

**Table 6:** Transcription rates of genes involved in cell structure^a^

*Hfx. alexandrinus* DSM 27206 ORF^b^	Gene name	Log_2_FC			Annotation
		0.1 mM	0.25 mM	0.5 mM	
Hfx-2019-SC1-cds84		-	4.64	-	SHCOT domain-containing membrane protein
Hfx-2019-SC1-cds278	*bmpA*	-	-	2.44	Basic membrane protein A
Hfx-2019-SC5-cds185		-	-	2.21	S-layer domain-containing protein
Hfx-2019-SC4-cds59		-	-3.43	-	PGF-CTERM sorting domain-containing protein

^a^RNA-Seq was used to determine the expression levels of the genes in *Hfx. alexandrinus* DSM 27206, as detailed in the section on Materials and Methods. The data are the average of three replicates. The threshold of differential expression genes was: |log_2_(FoldChange)|> 1 and qvalue < 0.00. Dashes indicate that there were minimal changes to the transcription, not considered statistically significant. ^b^The gene name, number, and annotation were generated through the gDNA sequencing of the tested strain, as described in the Materials and Methods section

At 0.25 mM silver, the expression of an archaea-specific PGF-CTERM domain-containing protein (Hfx-2019-SC4-cds59) was significantly suppressed (Table 6). This sorting signal is recognized by an archaeosortase and cleaved by a cysteine peptidase during the synthesis of the main cell surface glycoprotein in *Hfx. volcanii* [108, 109].

### Cell signaling

At 0.5 mM silver, the response regulator Hfx-2019-SC2-cds1096 was underexpressed by -2.15 log_2_FC (Table 7). Both in bacteria and archaea, the two-component signal transduction cascades comprise a membrane-localized sensor kinase that, upon stimulation, transfers a phosphate group to a soluble response regulator, resulting in a transcription-level cellular response [110, 111]. This downregulation may assist in preserving a well-balanced cellular response to silver-stress by suppressing energetically costly gene expression. Moreover, the putative TatA Sec-independent protein secretion pathway component (Hfx-2019-SC2-cds559), and a probable Tat-secreted protein containing the signal sequence of the Twin-arginine translocation pathway (Hfx-2019-SC1-cds229) were downregulated during cultivation with 0.1 mM silver nitrate (Table 7).

**Table 7:** Transcription rates of genes involved in cell signaling^a^

*Hfx. alexandrinus *DSM 27206 ORF^b^	Gene name	Log_2_FC			Annotation
		0.1 mM	0.25 mM	0.5 mM	
Hfx-2019-SC1-cds229		-2.04	-	-	Twin-arginine translocation pathway signal sequence-containing protein
Hfx-2019-SC2-cds559		-2.16	-	-	TatA Sec-independent protein secretion pathway component
Hfx-2019-SC2-cds1096		-	-	-2.15	Response regulator, His Kinase A (phospho-acceptor) domain
Hfx-2019-SC5-cds196		-	-2.41	-	Protein containing a halocin C8-like bacteriocin domain

^a^RNA-Seq was used to determine the expression levels of the genes in *Hfx. alexandrinus* DSM 27206, as detailed in the section on Materials and Methods. The data are the average of three replicates. The threshold of differential expression genes was: |log_2_(FoldChange)|> 1 and qvalue < 0.00. Dashes indicate that there were minimal changes to the transcription, not considered statistically significant. ^b^The gene name, number, and annotation were generated through the gDNA sequencing of the tested strain, as described in the Materials and Methods section

### RT-qPCR assessment

**Fig. 9:**
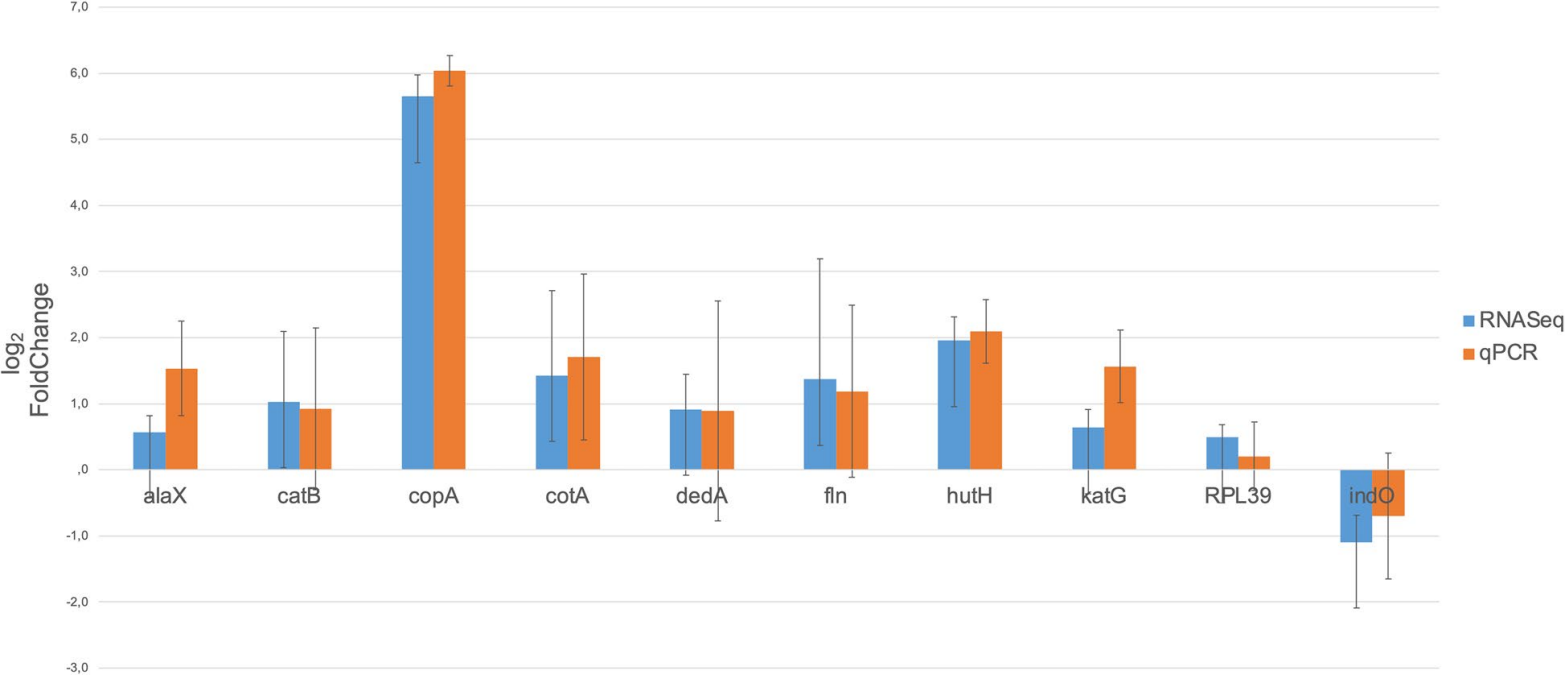
RT-qPCR validation of RNA-Seq data. RNA-Seq and RT-qPCR measurements of the transcriptional changes in particular genes are represented by rectangles. The standard deviations of three replicates are shown in bars

In order to validate the RNA-Seq findings, the expression levels of 10 genes were assessed using RT-qPCR, in triplicate. Both RT-qPCR and RNAseq analyses consistently revealed similar expression patterns for each of the examined genes (Fig. 9). Moreover, the calculation of the Pearson’s correlation coefficient revealed a robust correlation in the observed fold changes of the tested genes (R^2^ = 0.93; see Additional file 1). Consequently, the RT-qPCR results confirm the reliability and consistency of the RNA-Seq data, emphasizing the capability of RNA-Seq experiments to identify candidate genes responsive to elevated levels of heavy metals. Nevertheless, it is important to note that previous studies have indicated the absence of a universally ideal housekeeping gene and the potential influence of metal stress on the transcription of any protein-coding gene [112, 113]. Therefore, while RT-qPCR validation is valuable, it should be considered that it has certain limitations, and further refinements may be necessary to enhance the accuracy of this method.

## Conclusions

RNA-Seq profiling was employed to compare transcriptomes of *Hfx. alexandrinus* cells subjected to silver stress. We demonstrated that the investigated strain was able to elicit a tailored response to the presence of increasing concentrations of silver salts, primarily involving the differential expression of genes associated with metal transporters, basic metabolism, oxidative stress, and cellular motility. Notably, our findings underscore the vital role of the CopA copper ATPase in the rapid response system that regulates the movement of metal ions within cells. Furthermore, cells adapt to the adverse environmental conditions by undergoing a series of metabolic shifts, enabling them to efficiently redistribute their available resources while simultaneously engaging a variety of stress-response mechanisms.

The elucidation of the specific cellular response to toxic levels of silver ions in extremely halophilic *Haloferax alexandrinus*, as investigated through RNA-Seq analysis, confers unprecedented insights into the cellular components and mechanisms responsible for heavy-metal tolerance in haloarchaea.

While *Hfx. volcanii* has been commonly used as an archaeal model in various molecular genetics studies, the question of whether this model organism shares genes involved in silver stress response with substantial homology to those in *Hfx. alexandrinus* remains to be explored. Nonetheless, conducting additional comparative genome-wide studies on the 23 currently documented species within the *Haloferax* genus [114], could provide valuable insights into the evolutionary aspects of heavy metal stress response in halophilic archaea of the *Halobacteria* class.

### Supplementary Information

**Additional file 1.**

## Data Availability

The reference genome of *Hfx. alexandrinus* DSM 27206 assembled in this study is available in the National Center for Biotechnology Information (NCBI) repository under accession number ASM3053521v1. This genomic data is linked to Project Accession PRJNA986534 and Sample Accession SAMN25841318. https://www.ncbi.nlm.nih.gov/bioproject/986534 The raw RNA-Seq data generated for this study were deposited in the NCBI Short Read Archive database (SRA) under the accession numbers SRX20759387 through SRX20759398. Additionally, associated BioProject PRJNA986818 and BioSamples SAMN35848578 through SAMN35848589 are available for reference. https://www.ncbi.nlm.nih.gov/bioproject/PRJNA986818
