# Supplementary material for: Genome-wide transcriptional response to silver stress in extremely halophilic archaeon Haloferax alexandrinus DSM 27206 T
Source: BMC Microbiol. 2023 Dec 4;23:381. doi: 10.1186/s12866-023-03133-z (PMC10694973; doi:10.1186/s12866-023-03133-z)
Supplement: Supplementary file 1 — Additional file 1. [file 12866_2023_3133_MOESM1_ESM.docx]

| **Supplementary Table 1.** Selected transcripts and their corresponding primer pairs used in RT-qPCR analysis | | | |
| --- | --- | --- | --- |
| **ORF** | **Gene name** | **Sequence** | **Fragment Size (bp)** |
| - | *16s-Hfx** | 5‘-CGTCCGCAAGGATGAAA-3’ | 145 |
|  |  | 5‘-CAGCGTCGTGGTAAGG -3’ |  |
| Hfx-2019-SC1-cds4 | *copA* | 5‘-GGTGAACCAAAACGGCGTC-3’ | 120 |
|  |  | 5‘-AGGTTCTGAATCTCGGGCTG-3’ |  |
| Hfx-2019-SC4-cds58 | *cotA* | 5‘-AGTTCCAAGAGTCGTTCGCC-3’ | 149 |
|  |  | 5‘-GTCGTCGATGAGGTCGTAGTC-3’ |  |
| Hfx-2019-SC4-cds282 | *fln* | 5‘-ATGTCCGACTCGGCGTTC-3’ | 150 |
|  |  | 5‘-TTTGAGCCAGTTACACGCCT-3’ |  |
| Hfx-2019-SC1-cds34 | *katG* | 5‘-AGTTCCAAGAGTCGTTCGCC-3’ | 149 |
|  |  | 5‘-GTCGTCGATGAGGTCGTAGTC-3’ |  |
| Hfx-2019-SC6-cds193 | *RPL39* | 5‘-TCGAAGTCCAAGAAGAAGCGT -3’ | 101 |
|  |  | 5‘-GGGTTTCGCGTGACTTCCAT -3’ |  |
| Hfx-2019-SC4-cds57 | *dedA* | 5‘-CACAGCCGTTCCAGTAGTCA -3’ | 141 |
|  |  | 5‘-AGGTCGTTTCGGCGATACTC -3’ |  |
| Hfx-2019-SC4-cds202 | *indO* | 5‘-GCAGAACGCCGACACCTA-3’ | 146 |
|  |  | 5‘-CACCAGTCACGAACGAAGAC-3’ |  |
| Hfx-2019-SC2-cds233 | *alaX* | 5‘-ACGACACACGAGTTCGAGG-3’ | 128 |
|  |  | 5‘-CCGTCTACCGATAGGGTGC-3’ |  |
| Hfx-2019-SC5-cds98 | *hutH* | 5‘-GTCCTCGAATCGCACAAGGA -3’ | 141 |
|  |  | 5‘-GTCGGTGACGCTGTTGAGTT -3’ |  |
| Hfx-2019-SC4-cds21 | *catB* | 5‘-CCTGCAGTACCTCGAACAGC -3’ | 134 |
|  |  | 5‘-CCTCGACGAGCGAAGAGATG -3’ |  |
| **Haloferax*-specific 16s rRNA primers (Cai *et al.*, 2014). | | | |

| **Supplementary Table 2.** Overview of the mapping status of the RNA-Seq reads to the reference genome of *Hfx. alexandrinus* DSM 27206. Biological triplicates of the samples are included, as follows: Ha_C_1, Ha_C_2, and Ha_C_3- control cultures; Ha_01_1, Ha_01_2, and Ha_01_3- cells cultured with 0.1 mM silver nitrate; Ha_025_1, Ha_025_2, and Ha_025_3- cells cultured with 0.25 mM silver nitrate; Ha_05_1, Ha_05_2, and Ha_05_3- cells cultured with 0.5 mM silver nitrate. |
| --- |
| \| **Sample name** \| **Ha_C_1** \| **Ha_C_2** \| **Ha_C_3** \| **Ha_01_1** \| **Ha_01_2** \| **Ha_01_3** \| **Ha_025_1** \| **Ha_025_2** \| **Ha_025_3** \| **Ha_05_1** \| **Ha_05_2** \| **Ha_05_3** \| \| --- \| --- \| --- \| --- \| --- \| --- \| --- \| --- \| --- \| --- \| --- \| --- \| --- \| \| Total reads \| 22176134 \| 21831688 \| 21470938 \| 18029000 \| 14607204 \| 14093276 \| 17111078 \| 16110356 \| 14394464 \| 16330482 \| 15060428 \| 13614582 \| \| Total mapped reads \| 18839884 \| 18853893 \| 17886518 \| 16149300 \| 12062943 \| 11029258 \| 12682770 \| 12653390 \| 11430059 \| 13779482 \| 12913814 \| 11296498 \| \| Uniquely mapped reads \| 18491880 \| 18517629 \| 17571517 \| 15886396 \| 11837644 \| 10813443 \| 12474792 \| 12450181 \| 11244505 \| 13575051 \| 12718173 \| 11128384 \| \| Multiple mapped reads \| 348004 \| 336264 \| 315001 \| 262904 \| 225299 \| 215815 \| 207978 \| 203209 \| 185554 \| 204431 \| 195641 \| 168114 \| \| Total mapping rate \| 84.96% \| 86.36% \| 83.31% \| 89.57% \| 82.58% \| 78.26% \| 74.12% \| 78.54% \| 79.41% \| 84.38% \| 85.75% \| 82.97% \| \| Uniquely mapping rate \| 83.39% \| 84.82% \| 81.84% \| 88.12% \| 81.04% \| 76.73% \| 72.90% \| 77.28% \| 78.12% \| 83.13% \| 84.45% \| 81.74% \| \| Multiple mapping rate \| 1.57% \| 1.54% \| 1.47% \| 1.46% \| 1.54% \| 1.53% \| 1.22% \| 1.26% \| 1.29% \| 1.25% \| 1.30% \| 1.23% \| |
| \| 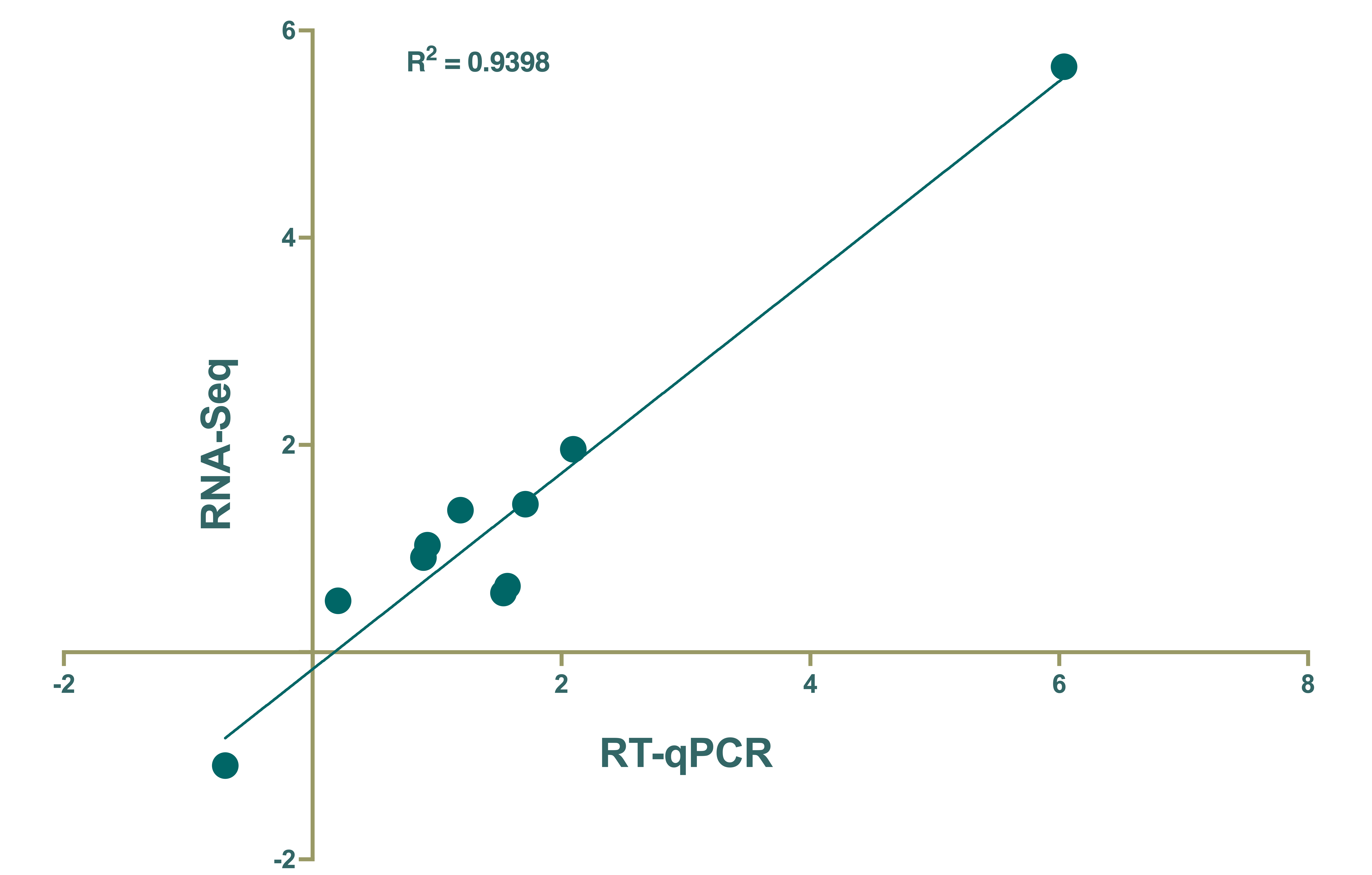 \| \| --- \| \| **Supplementary Figure 1:** Correlation plot (Pearson’s correlation coefficient) between fold changes  of selected genes observed in RNA-seq and their corresponding fold-change values in RT-qPCR analysis. \| |
